# A novel m6A/m5C/m1A score signature to evaluate prognosis and its immunotherapy value in colon cancer patients

**DOI:** 10.1007/s00432-023-05033-1

**Published:** 2023-07-08

**Authors:** Jinsong Liu, Min Dou, Xiuling Liu, Yueyao Lu, Wenbin Lu

**Affiliations:** 1 Department of Oncology, Wujin Hospital Affiliated With Jiangsu University, Changzhou, 213017 Jiangsu China; 2 Wujin Institute of Molecular Diagnostics and Precision Cancer Medicine, Jiangsu University, Changzhou, 213017 Jiangsu China; 3 Changzhou Key Laboratory of Molecular Diagnostics and Precision Cancer Medicine, Changzhou, 213017 Jiangsu China; 4 Bengbu Medical College, Bengbu, 233000 Anhui China; 5 Department of Oncology, Changzhou Clinical Medical College, Nanjing Medical University, Changzhou, 213017 Jiangsu China; 6 Department of Oncology, The Wujin Clinical College of Xuzhou Medical University, Changzhou, 213017 Jiangsu China

**Keywords:** m6A/m5C/m1A, Prognosis, Immunotherapy value, Colon cancer

## Abstract

**Background:**

Colon cancer features strong heterogeneity and invasiveness, with high incidence and mortality rates. Recently, RNA modifications involving m6A, m5C, and m1A play a vital part in tumorigenesis and immune cell infiltration. However, integrated analysis among various RNA modifications in colon cancer has not been performed.

**Methods:**

RNA-seq profiling, clinical data and mutation data were obtained from The Cancer Genome Atlas and Gene Expression Omnibus. We first explored the mutation status and expression levels of m6A/m5C/m1A regulators in colon cancer. Then, different m6A/m5C/m1A clusters and gene clusters were identified by consensus clustering analysis. We further constructed and validated a scoring system, which could be utilized to accurately assess the risk of individuals and guide personalized immunotherapy. Finally, m6A/m5C/m1A regulators were validated by immunohistochemical staining and RT-qPCR.

**Results:**

In our study, three m6A/m5C/m1A clusters and gene clusters were identified. Most importantly, we constructed a m6A/m5C/m1A scoring system to assess the clinical risk of the individuals. Besides, the prognostic value of the score was validated with three independent cohorts. Moreover, the level of the immunophenoscore of the low m6A/m5C/m1A score group increased significantly with CTLA-4/PD-1 immunotherapy. Finally, we validated that the mRNA and protein expression of VIRMA and DNMT3B increased in colon cancer tissues.

**Conclusions:**

We constructed and validated a stable and powerful m6A/m5C/m1A score signature to assess the survival outcomes and immune infiltration characteristics of colon cancer patients, which further guides optimization of personalized treatment, making it valuable for clinical translation and implementation.

**Supplementary Information:**

The online version contains supplementary material available at 10.1007/s00432-023-05033-1.

## Introduction

Colon cancer is gastrointestinal tumor featuring obvious heterogeneity and aggressiveness (Tauriello et al. 2017). According to global cancer statistics, there are 1,148,515 new individuals and 576,858 deaths related to colon cancer, and colon cancer ranks fifth in incidence and mortality rate among all cancers (Sung et al. 2021). Although there has been accelerated progress in diagnosis and treatment technology, including colonoscopy screening and surgical procedures, the 5-year survival rates of patients with colon cancer remain low (Liu et al. 2022b). Many colon cancers are already in advanced stages when they are detected. The main reasons for this are the high cost of colon cancer screening and the lack of sensitive screening indicators (He et al. 2022). Therefore, constructing a clinical signature to describe the characteristics of colon cancer and predict the overall survival of patients is imperative to further develop individualized treatment approaches.

There are different types of epigenetic variations, including chromatin reshaping, noncoding RNA (long noncoding RNAs and microRNAs) effects, and RNA modification, which are involved in key signaling pathways and can act as clinically relevant biomarkers in cancers (Jung et al. 2020). Currently, more than 170 kinds of modification types have been found in intracellular RNA, including mRNAs, tRNAs, rRNAs, and ncRNAs (Shi et al. 2020). These modifications contribute to pre-mRNA splicing, export, and stability. There are different kinds of RNA modifications, including N6-methyladenosine (m6A), 5-methylcytosine (m5C), and N1-methyladenosine (m1A) (Xie et al. 2020). These modifications are mediated by regulators including writers, readers, and erasers. The most well characterized RNA modification is m6A. The methyltransferase-like METTL3/METTL14 complex (writers) plays a catalytic role in the deposition of m6A on mRNA (Wang et al. 2016a; Wang et al. 2016b). M6A can be reversed by two different RNA demethylases: FTO and ALKBH5 (Jia et al. 2011; Zheng et al. 2013). In addition, m6A is recognized by various reader proteins, including the YTH domain families (Li et al. 2014). Similar to DNA, RNA modifications could occur at position 5 of cytidine residues, which is known as m5C (Edelheit et al. 2013). The functions of m5C vary in various RNA types. In tRNA, m5C modulates the stability and organization of RNA, contributing to translation accuracy. However, in rRNA, loss of methylcytosine maintains translational readthrough of stop codons. Most importantly, in mRNA, the methyltransferases of m5C are the NSUN family proteins (Yang et al. 2017). The Aly/REF export factor (ALYREF) serves as a reader, maintaining the nuclear export of mature mRNAs (Bohnsack et al. 2019). RNA demethylases of m5C are members of the tet methylcytosine dioxygenase family. Recently, there has been an increased focus on m1A, generated by methylation of adenosine at position 1 (Dominissini et al. 2016). The TRM6/61 complex is a methyltransferase catalyzing m1A formation (Chujo and Suzuki 2012; Vilardo et al. 2012). Similar to m6A, YTH family members can regulate m1A by binding to mRNA sites (Dai et al. 2018). In addition, m1A is erased from tRNAs by ALKBH1 and ALKBH3 and mRNA only by ALKBH3 (Liu et al. 2016). There is a close correlation between m1A and cell proliferation in gastrointestinal cancers. Consequently, m6A, m5C, and m1A regulators in colon cancer are worth further exploration.

However, previous studies have only investigated single RNA modifications, whose established signatures have some limitations owing to various modifications interacting with each other and working together in colon cancer (Qi et al. 2022). Therefore, we assessed cross-talk among various RNA modifications to construct a novel scoring system that evaluates the survival of patients, further paving the way for optimizing personalized treatment. In our work, we first investigated mutation traits of 48 m6A/m5C/m1A regulators in colon cancer. Next, we identified m6A/m5C/m1A clusters using unsupervised cluster analysis. Survival analysis, immune infiltration analysis, and biological function exploration were performed among different m6A/m5C/m1A clusters. Then, we used differential analysis and univariate Cox analysis to obtain the prognostic differentially expressed genes (DEGs). Moreover, gene clusters were constructed, and the corresponding analysis was also performed in different gene clusters. Considering the differential expression and complexity of m6A/m5C/m1A regulators, we constructed a scoring system and validated the prognostic value of the score with three independent cohorts. Subsequently, we analyzed survival, immune infiltration, tumor mutation status, clinical characteristic and the immunotherapy response. Finally, we validated m6A/m5C/m1A regulators at the protein and mRNA expression levels, which further identified that VIRMA and DNMT3B played vital roles in colon cancer.

## Materials and methods

### Data collection

RNA-seq transcription profiles, clinical data and mutation data were obtained from TCGA (https://portal.gdc.cancer.gov/). 473 colon cancer samples and 41 normal tissue samples were obtained. Gene expression profiles of GSE39582 dataset were obtained from GEO (https://www.ncbi.nlm.nih.gov/geo/). GSE39582 dataset contains 566 cancer samples and 19 normal samples, follow-up survival analysis and model validation are not required for normal samples, so they should be excluded. To process gene expression data from the TCGA and GSE39582, which were measured by different platforms, the Fragments Per Kilobase of exon model per Million mapped fragments (FPKM) format data were downloaded using the “fpkm” function in R to convert them to the transcripts per kilobase million (TPM) for the next analysis (Wagner et al. 2012). For validating the score efficacy, the following three GEO cohorts were employed: GSE29621 (n = 65), GSE17536 (n = 177), and GSE17537 (n = 55). The above cohorts with complete patient information including clinicopathological parameters and follow-up records. Then, the copy number variation (CNV) of colon cancer patients was abstracted from UCSC Xena (http://xena.ucsc.edu/) (Goldman et al. 2020). We collected 23 m6A, 15 m5C, and 10 m1A regulators from the previous literature (Haruehanroengra et al. 2020; Song et al. 2022). Detailed information on the m6A/m5C/m1A regulators is shown in Supplemental Table 1.

### M6A/m5C/m1A regulators in the cancer genome map

To understand the functional mechanism of m6A/m5C/m1A regulators more clearly, we first constructed a functional pattern map of m6A/m5C/m1A regulators in RNA metabolism. Next, the mutation trait, CNV data, gene location, expression levels, and interaction relationships of 48 m6A/m5C/m1A regulators in colon cancer were analyzed with the following R packages: maftools, RCircos, limma, reshape2, ggpubr, igraph, psych, and RColorBrewer.

### Survival analysis of m6A/m5C/m1A regulators in colon cancer patients

The predictive values of m6A/m5C/m1A regulators in colon cancer were assessed using Kaplan–Meier (K-M) and univariate Cox regression analyses. The R packages “survival” and “survminer” were used in the process. The results from these analyses are shown with the K–M curve.

### Consensus clustering analysis of m6A/m5C/m1A regulators and corresponding functional analysis

Cluster clustering analysis is a method of subsampling from a set of sample data (e.g., a microarray) and identifying clusters with a specified number of clusters (k). For each k, pairwise consistency values, i.e., the proportion of occurrences of two samples in the same subsample to the same cluster, are calculated and stored in a symmetric consensus matrix. We performed unsupervised clustering analysis with the R package “ConsensusClusterPlus” (Wilkerson and Hayes 2010). The optimal classification was identified based on the cumulative distribution function (CDF) curve and the correlations between clusters. The overall survival (OS) of m6A/m5C/m1A clusters was assessed by K–M curves. Moreover, to analyze the levels of immune cells in tumor samples among m6A/m5C/m1A clusters, we performed single-sample gene set enrichment analysis (ssGSEA) with the R package “GSEABase” (Hanzelmann et al. 2013). The heatmap was constructed with the R package “pheatmap”, and the differences were identified using the R package “limma”. To further analyze the function of m6A/m5C/m1A clusters in biological pathways, we utilized gene set variation analysis (GSVA) (Foroutan et al. 2018).

### Analysis of DEGs among m6A/m5C/m1A clusters

We first obtained m6A/m5C/m1A regulator-related DEGs between different m6A/m5C/m1A clusters with the R package “limma, VennDiagram” (Ritchie et al. 2015), and the significance filtering criterion was an adjusted *p* value < 0.001. To explore potential molecular functions and associated biological pathways, we analyzed m6A/m5C/m1A regulator-related DEGs with GO and KEGG analysis by the R packages “clusterProfiler” and “enrichplot” (Yu et al. 2012). The above results were visualized with bubble charts. Next, we further utilized univariate Cox analysis to obtain the prognostic DEGs. Then, the consensus clustering method was used to identify proper gene clusters, and a K–M curve was constructed to assess the OS for patients in different gene clusters. Finally, we also analyzed the levels of m6A/m5C/m1A regulators among different gene clusters and generated a heatmap to explore the relationship between clinical characteristics and gene clusters.

### Construction and validation of the m6A/m5C/m1A scoring system by principal component analysis (PCA)

Based on the above results, we obtained the prognostic DEGs associated with m6A/m5C/m1A clusters. Then, we used PCA to analyze the prognostic DEGs, thus yielding the m6A/m5C/m1A score system. The m6A/m5C/m1A score was calculated with the following formula: m6A/m5C/m1Ascore = (PC1i + PC2 i), where i represents the levels of m6A/m5C/m1A-related genes (Sotiriou et al. 2006; Zeng et al. 2019). Principal components 1 and 2 were obtained to construct the m6A/m5C/m1A score (Sotiriou et al. 2006). A Sankey diagram was constructed to explore the relationship among m6A/m5C/m1A clusters, gene clusters, m6A/m5C/m1A scores, and fustats. Moreover, the Kaplan‒Meier curve was constructed and validated to assess the OS for the m6A/m5C/m1A score, and correlation analysis between the m6A/m5C/m1A scores and immune cells was explored with the R package “corrplot”. We also investigated the levels of the m6A/m5C/m1A score in different m6A/m5C/m1A clusters and gene clusters with the Kruskal–Wallis test.

### Differences in clinical traits and immunotherapy among the m6A/m5C/m1A score groups

We first analyzed the mutation characteristics of colon cancer patients in different m6A/m5C/m1A score groups using the waterfall chart with the R package “maftools”. Moreover, K–M analysis was performed to investigate the differences in survival outcomes and clinical characteristics (age, sex, stage) between different score groups. Considering the clinical value of tumor mutation burden (TMB) in providing a theoretical direction for immunotherapy (Mariathasan et al. 2018), stratified survival analysis was used to explore the correlation between TMB and the score. In terms of the response to anti-CTLA-4 and anti-PD-1, immunophenoscore (IPS) is a reliable predictor (Charoentong et al. 2017). There were four sample types, including CTLA-4 (pos/neg)/PD-1 (pos/neg), according to the IPS. Pos and neg indicate positive expression and negative expression, respectively. Therefore, we explored the IPS in the m6A/m5C/m1A score groups with the R package “ggpubr”. Finally, we also analyzed the m6A/m5C/m1A score in samples with different MSI types.

### Validation of m6A/m5C/m1A regulators with the human protein atlas and real-time quantitative PCR (RT-qPCR)

The data, including immunohistochemical staining data for colon cancer and normal tissues, were obtained from the HPA (http://www.proteinatlas.org/) (Liu et al. 2022a). We first validated differentially expressed m6A/m5C/m1A regulators at the protein expression level. There were four degrees of staining: high, medium, low, and not detected. Finally, we further validated hub m6A/m5C/m1A regulators at the mRNA expression level with RT-qPCR. TRIzol reagent (TaKaRa) was used to extract RNA based on the corresponding protocol. Reverse transcription was then performed with a TaqMan cDNA synthesis kit (TaKaRa). The SYBR Green PCR Kit (TaKaRa) was used for RT‒qPCR. The detailed experimental procedure has been described previously (Zeng et al. 2022). The main sequences are shown in Supplemental Table 2. The 2^−ΔΔCt^ algorithm was performed to calculate gene expression levels. Ten pairs of colon cancer and normal colon tissues were provided by Wujin Hospital Affiliated with Jiangsu University.

### Statistical analysis

Statistical analysis was performed using R 3.6.3 software or GraphPad Prism 9.0. The Wilcoxon and Kruskal–Wallis test were used for different group comparisons. Log-rank tests were employed in the Kaplan–Meier analysis. Spearman’s test was used in the immune cell infiltration analysis. A *p* value < 0.05 was considered statistically significant. *p < 0.05, **p < 0.01, ***p < 0.001. All experiments were repeated three times independently.

## Results

### The expression and variation of m6A/m5C/m1A regulators in colon cancer

**Fig. 1:**
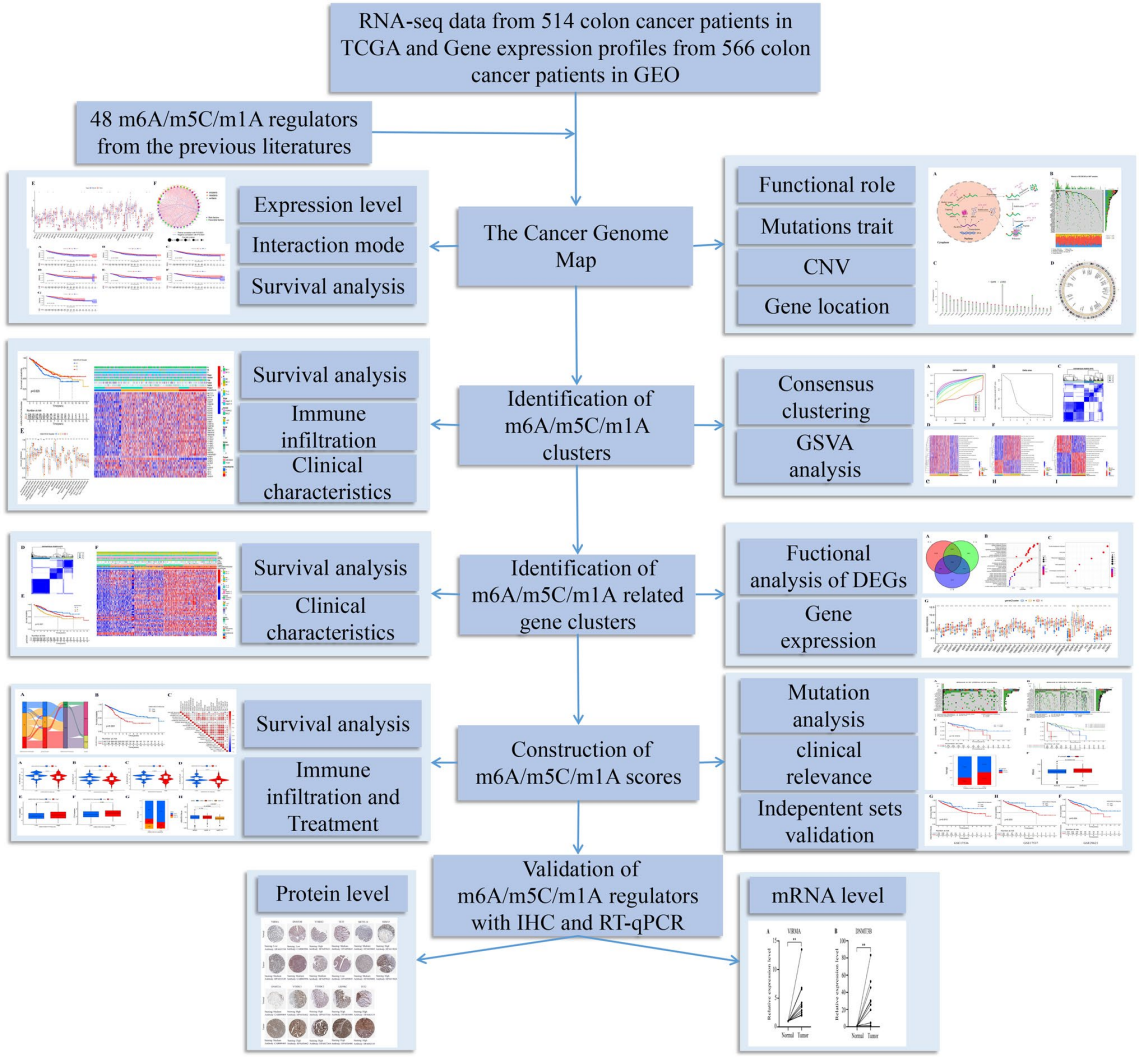
Flow chart of our work

**Fig. 2:**
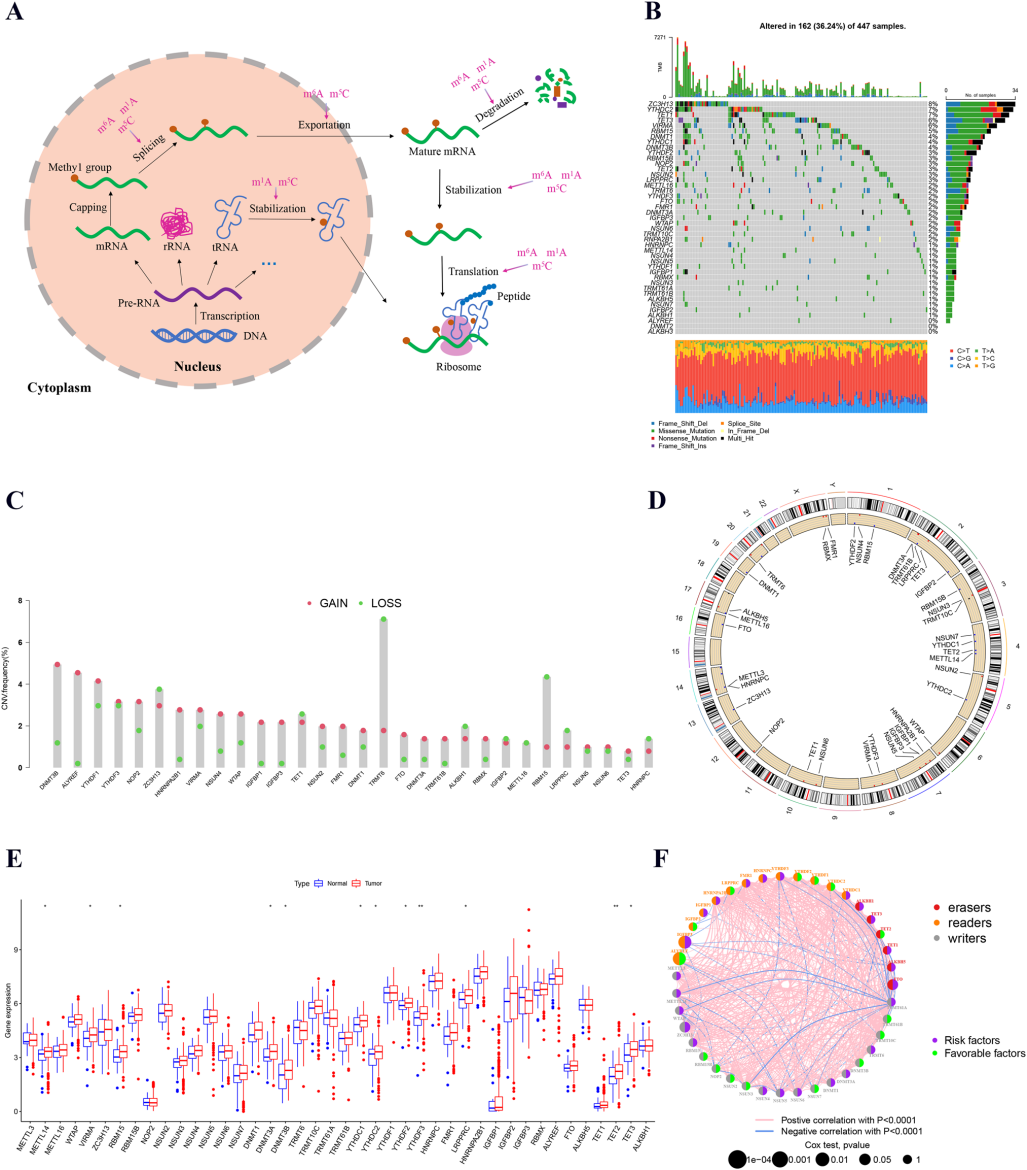
The expression and variation of m6A/m5C/m1A regulators in colon cancer. **A** The functional mode of m6A/m5C/m1A regulators in RNA metabolism. **B** The mutation rate of m6A/m5C/m1A regulators. Different colors represent different types of mutations. **C** Copy number variation of m6A/m5C/m1A regulators in colon cancer patients. The column indicates the variation frequency. Red and green circles represent amplification and deletion frequencies, respectively. **D** The positions of CNVs in m6A/m5C/m1A regulators on human chromosomes. Different colors represent different chromosomes. **E** The levels of m6A/m5C/m1A regulators in colon cancer tissues and normal tissues. Red and blue indicate tumor and normal tissues, respectively. **F** The expression interaction of m6A/m5C/m1A regulators in colon cancer patients. The size of each circle corresponds to the *p* value. Left: red, orange, and gray represent modification types (erasers, readers, and writers, respectively). Right: green and purple represent favorable factors and risk factors, respectively. *p < 0.05, **p < 0.01, ***p < 0.001

A flow chart of our work was shown in Fig. 1. In our study, we first obtained 48 m6A/m5C/m1A regulators from a previous study. We constructed a functional pattern map of m6A/m5C/m1A regulators in RNA metabolism, which helped us understand the functional mechanism of m6A/m5C/m1A regulators more clearly (Fig. 2A). From the pattern diagram, we concluded that m6A/m5C/m1A regulators interacted with each other and jointly participated in RNA metabolism, including the splicing, exportation, stabilization, degradation, and translation of various RNAs. Therefore, we studied the value of all RNA regulators, including m6A, m5C, and m1A regulators, rather than individual RNA regulators. After obtaining the corresponding mutation data, we found that 162 (36.24%) of the 447 samples showed mutations in m6A/m5C/m1A regulators (Fig. 2B). ZC3H13, YTHDC2, TET1, TET3, VIRMA, and RBM15 had high mutation rates (> 5%). The top three mutation types were as follows: missense mutation, multihit mutation and frame shift deletion. Moreover, CNV analysis implied that ZC3H13, TET1, TRMT6, ALKBH1, IGFBP2, METTL16, RBM15, LRPPRC and HNRNPC showed extensive copy number loss, while the rest of the m6A/m5C/m1A regulators showed widespread CNV amplification (Fig. 2C). The circos plot showed the location of CNVs for the m6A/m5C/m1A regulators on the chromosome (Fig. 2D). In addition, we further investigated the levels of m6A/m5C/m1A regulators in colon cancer and found that most m6A/m5C/m1A regulators had higher expression levels in colon cancer tissue versus normal tissue independent of CNV, and 12 m6A/m5C/m1A regulators that showed a statistically significant difference between normal and tumor tissue (Fig. 2E). Finally, the correlations among 48 m6A/m5C/m1A regulators and their prognostic value in colon cancer were found (Fig. 2F). The above results indicated that the complexity of m6A/m5C/m1A regulators and tumor heterogeneity played vital roles in the occurrence and progression of colon cancer.

### The overall survival curve of m6A/m5C/m1A regulators in colon cancer patients

**Fig. 3:**
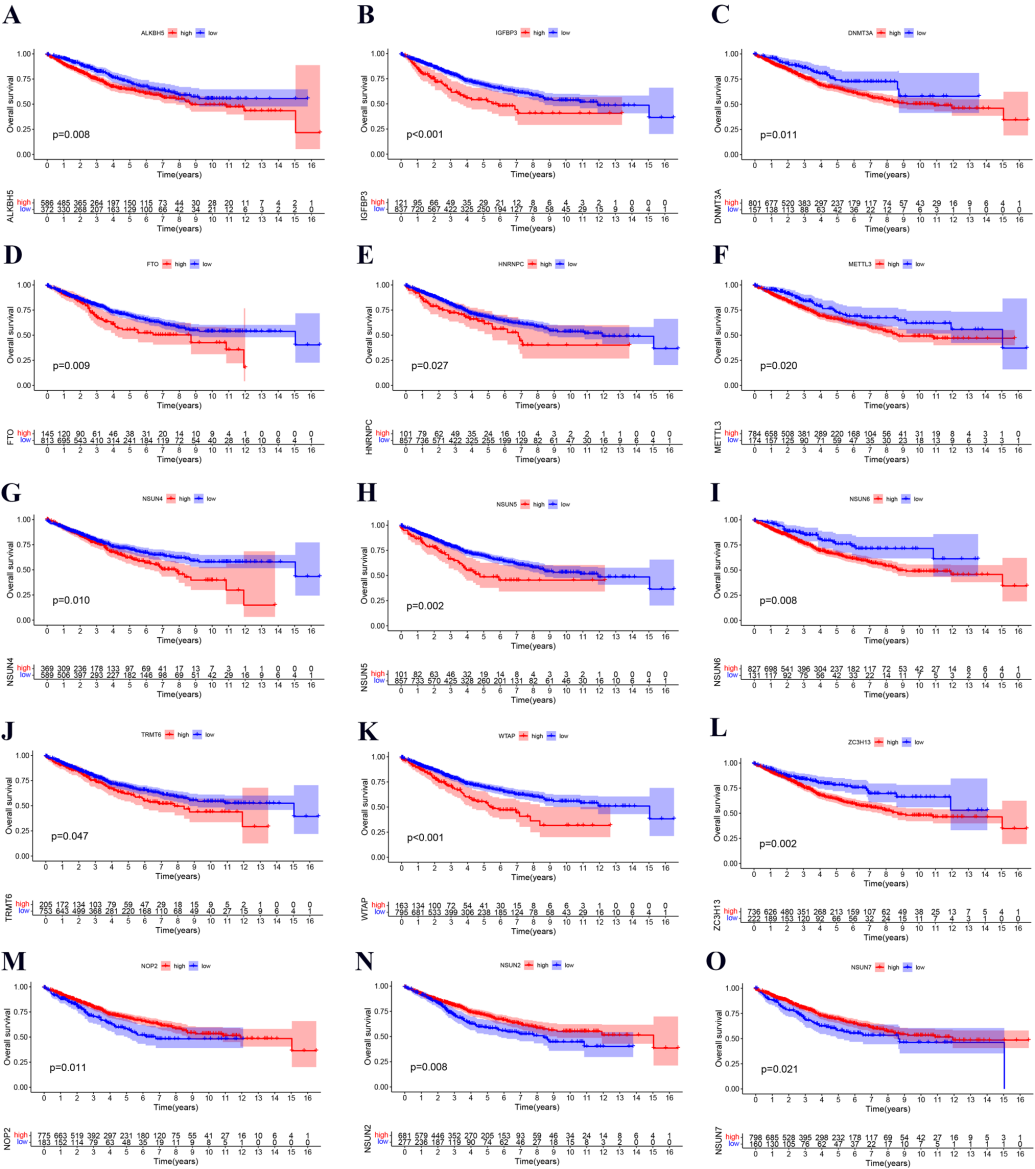
The overall survival curves of colon cancer patients treated with m6A/m5C/m1A regulators. **A**–**L** The low levels of 12 m6A/m5C/m1A regulators had a favorable prognosis. **M**–**O** High levels of 3 m6A/m5C/m1A regulators were associated with a favorable prognosis. Red and blue indicate high and low expression, respectively

To explore the predictive value of m6A/m5C/m1A regulators in colon cancer patients, we performed Kaplan‒Meier analysis. We found that low levels of m6A/m5C/m1A regulators, including ALKBH5, IGFBP3, DNMT3A, FTO, HNRNPC, METTL3, NSUN4, NSUN5, NSUN6, TRMT6, WTAP, and ZC3H13, indicated a favorable prognosis in colon cancer patients (Fig. 3), while high levels of m6A/m5C/m1A regulators, including NOP2, NSUN2, NSUN7, RBM15B, TET2, TRMT61B, ALYREF, LRPPRC, YTHDC2, and YTHDF1, indicated a favorable prognosis in colon cancer patients (Figure S1).

### Identification of m6A/m5C/m1A clusters

**Fig. 4:**
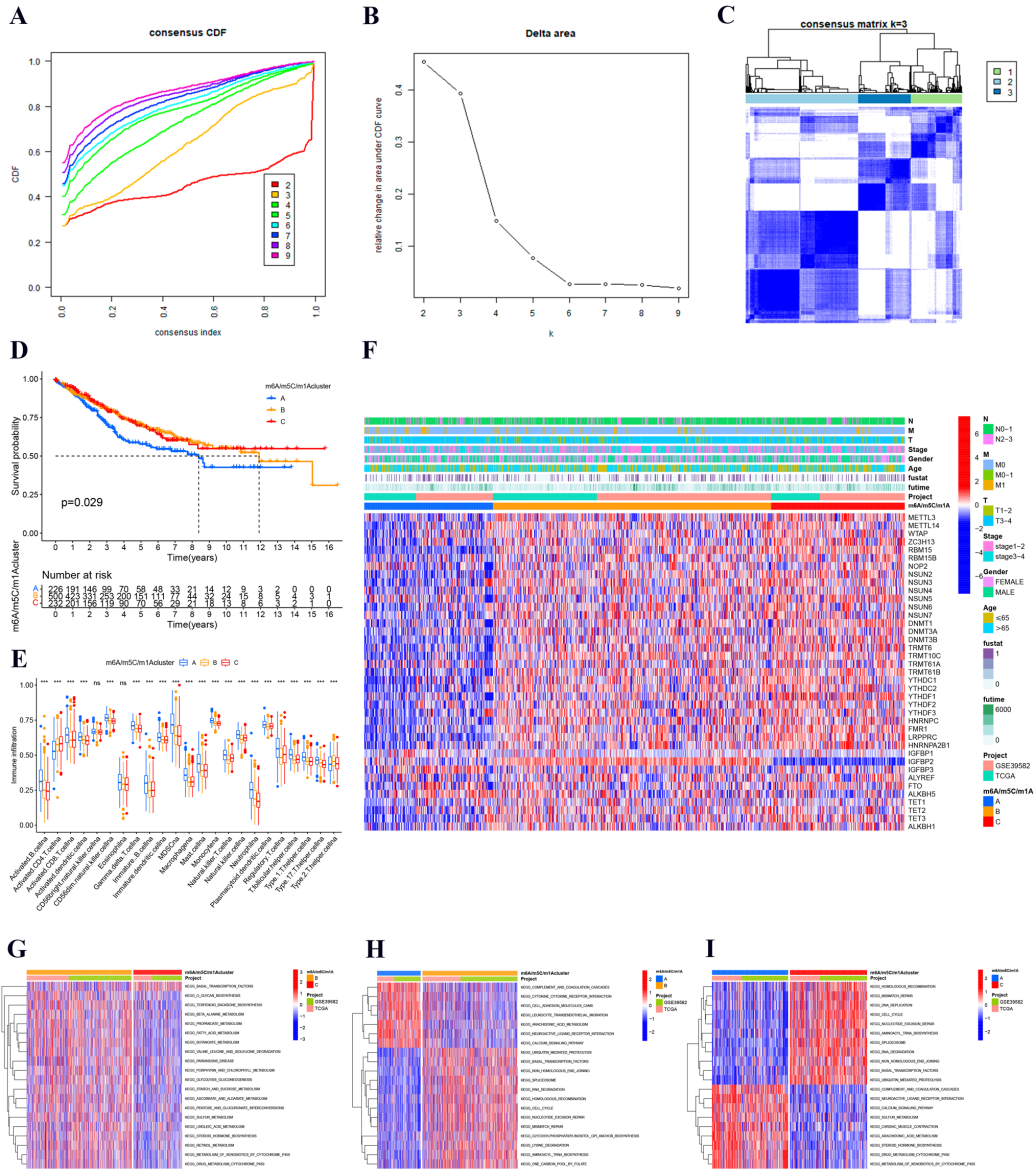
Identification of m6A/m5C/m1A clusters in colon cancer patients. **A**, **B** Cumulative distribution function curve and AUC in m6A/m5C/m1A cluster analysis from k = 2 to 9. AUC: Area under the curve. **C** Consensus clustering matrix at optimal k = 3. **D** The overall survival curve of colon cancer patients in three m6A/m5C/m1A clusters. Blue, yellow, and red represent clusters A, B, and C, respectively. **E** Differential levels of infiltrating immune cells among the three m6A/m5C/m1A clusters. **F** Heatmap indicating gene expression and clinical characteristics in three m6A/m5C/m1A clusters. Clinical characteristics included age, sex, and stage. **G**–**I** GSVA showing enrichment pathways among different clusters. *p < 0.05, **p < 0.01, ***p < 0.001

Three m6A/m5C/m1A clusters were classified with unsupervised clustering analysis. According to the relative area change of the CDF curve and the correlations between clusters, matrix k = 3 was the optimal number of clusters (Fig. 4A–C). Finally, three clusters were identified: m6A/m5C/m1A cluster A (n = 230), m6A/m5C/m1A cluster B (n = 504), and m6A/m5C/m1A cluster C (n = 233). According to the survival analysis, Fig. 4D shows that m6A/m5C/m1A cluster C had a better survival outcome, while m6A/m5C/m1A cluster A had a poor survival outcome (*p* = 0.029). Moreover, we further explored the differential levels of immune cells among the clusters. The results showed that the levels of most immune cells were elevated in the three m6A/m5C/m1A clusters and that the m6A/m5C/m1A cluster A had higher levels of immune cell infiltration than the other clusters (Fig. 4E). We also performed ssGSEA of immune functions and supplemented with the levels of other immune infiltrating cells in the three m6A/m5C/m1A clusters (Fig. S2). The above results better explained why m6A/ m5C/m1A cluster A leads to poorer survival outcome. In addition, a correlation heatmap, including the expression of m6A/m5C/m1A regulators, project, patient clinical characteristics (survival time, status, grade, stage, and age) and m6A/m5C/m1A clusters, was plotted. The results indicated that the expression of m6A/m5C/m1A regulators was higher in clusters B and C (Fig. 4F). Finally, to assess the biological functions of m6A/m5C/m1A regulators, we performed GSVA. The heatmap indicated that cluster A was mainly enriched in complement and coagulation cascades, cytokine receptor interaction, cell adhesion molecules cams, leukocyte transendothelial migration, arachidonic acid metabolism, and neuroactive ligand receptor interaction; cluster B was mainly enriched in homologous recombination, glycosylphosphatidylinositol GPI anchor biosynthesis, and lysine degradation; and cluster C was significantly enriched in basal transcription factors (Fig. 4G–I).

### Identification of m6A/m5C/m1A-related gene clusters

**Fig. 5:**
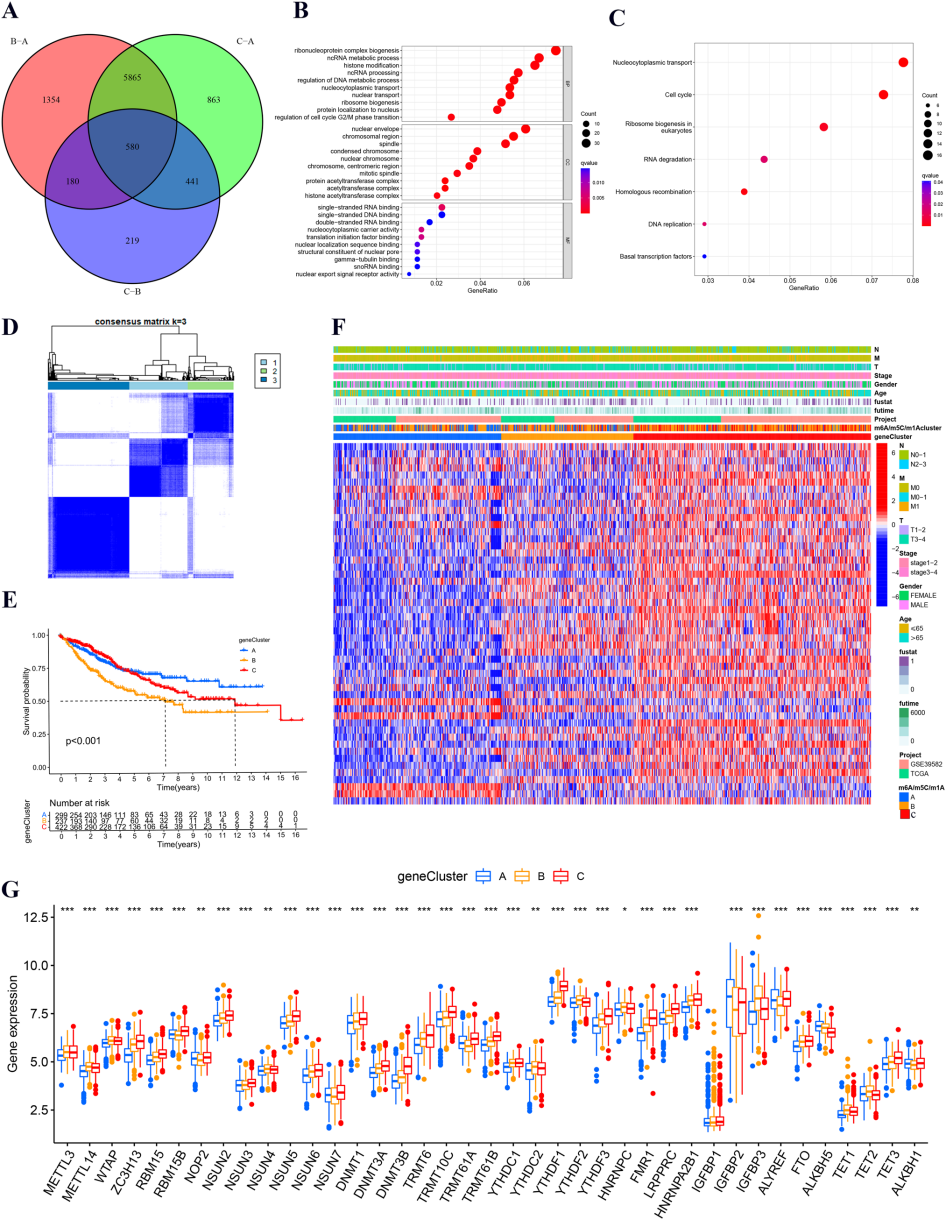
Identification of m6A/m5C/m1A-related gene clusters in colon cancer patients. **A** The overlapping m6A/m5C/m1A-related DEGs in a Venn diagram. **B**, **C** GO and KEGG analyses of overlapping DEGs. BP: biological process, CC: cellular component, MF: molecular function. The size of the circle represents the number of genes, and the color represents the different *p* values. **D** Consensus clustering matrix at optimal k = 3. **E** The overall survival curve of colon cancer patients in three gene clusters. **F** The heatmap visualized the correlation between clinical features of patients and different gene clusters. **G** Differential levels of m6A/m5C/m1A regulators among the three gene clusters

To further explore the clinical and biological traits of m6A/m5C/m1A clusters, a total of 580 DEGs were identified from the overlap across different m6A/m5C/m1A clusters in the Venn diagram (Fig. 5A). Then, GO results indicated that the DEGs mainly enriched in ribonucleoprotein complex biogenesis of biological process, nuclear envelope of cellular component, and single-stranded RNA binding of molecular function (Fig. 5B). KEGG results indicated that the DEGs mainly enriched in nucleocytoplasmic transport of the signaling pathway (Fig. 5C). Next, we further analyzed the DEGs of m6A/m5C/m1A clusters, and univariate Cox analysis was performed to obtain the prognostic DEGs. Then, the consensus clustering method was also used to identify proper gene clusters (k = 3), and there were three types of gene clusters: m6A/m5C/m1A-related gene cluster A (n = 304), cluster B (n = 238), and cluster C (n = 426) (Fig. 5D). Based on survival analysis, Fig. 5E shows that gene cluster A had a better survival outcome, while gene cluster B had a poorer survival outcome (*p* < 0.001). Figure 5F reveals that the three gene clusters had different clinical characteristics, and patients in gene cluster C showed an advanced clinical stage. Differential expression analysis indicated that the levels of most m6A/m5C/m1A regulators were elevated in the three gene clusters and that gene cluster C had higher levels of m6A/m5C/m1A regulators than the other gene clusters (Fig. 5G).

### Construction and validation of the m6A/m5C/m1A scoring system

**Fig. 6:**
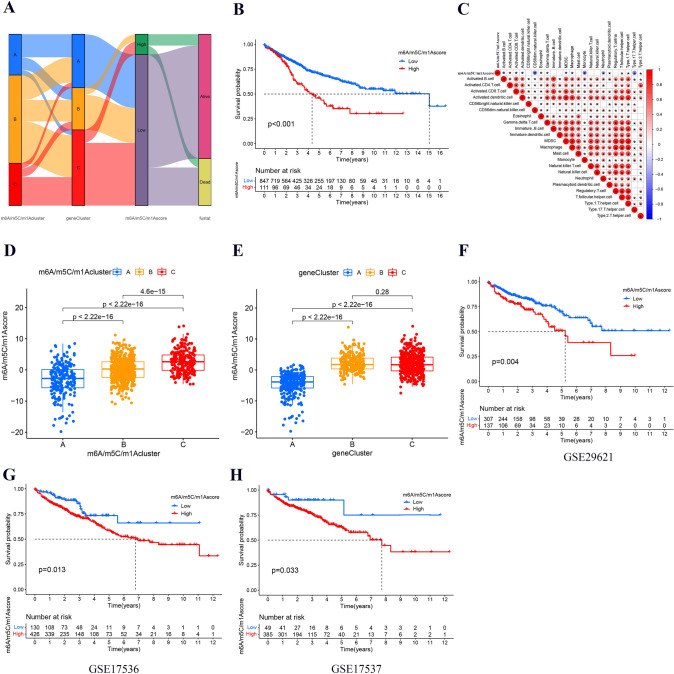
Construction and validation of the m6A/m5C/m1A score model. **A** Alluvial diagram showing the relationship among clusters, gene clusters, score groups, and fustats. **B** The overall survival curve of colon cancer patients in different score groups. **C** The correlations between m6A/m5C/m1A scores and infiltrating immune cells. **D**, **E** Differential levels of m6A/m5C/m1A scores in different clusters and gene clusters. Survival analysis of the patients with high and low m6A/m5C/m1A scores in the different colon cancer cohorts. **F** GSE29621 set, *p* = 0.004; **G** GSE17536 set,* p* = 0.013; **H** GSE17537 set, *p* = 0.033

Although m6A/m5C/m1A regulators play a vital role in regulating prognosis and immune infiltration, due to the differential expression and complexity of m6A/m5C/m1A regulators, the above analysis referred to these regulators on a broad scale rather than at the individual level. We constructed an m6A/m5C/m1A score system to evaluate the clinical value of m6A/m5C/m1A regulators in individuals. The alluvial diagram showed the relationship among clusters, gene clusters, score groups, and fustats, which indicated that m6A/m5C/m1A cluster B/C and gene cluster A/C mainly corresponded to low m6A/m5C/m1A scores (Fig. 6A). Figure 6B shows that the low m6A/m5C/m1A score group had a better survival outcome, while the high score group had a poorer survival outcome (*p* < 0.001). The above results were consistent with the previous survival outcomes observed for m6A/m5C/m1A clusters and gene clusters. Immune infiltration analysis indicated that the infiltration levels of activated CD4 T cells, eosinophils, plasmacytoid dendritic cells, and types 2T helper cells were positively connective with the m6A/m5C/m1A score, while the infiltration levels of the remaining immune cells were negatively connective with the m6A/m5C/m1A score (Fig. 6C). Higher m6A/m5C/m1A scores were observed in cluster B/C and gene cluster B (*p* < 0.001) (Fig. 6D, E). Next, the prognostic value of the score was validated in three independent cohorts. The results showed that the low m6A/m5C/m1A score group had a better survival outcome, while the high score group had a poorer survival outcome (GSE29621 set, *p* = 0.004; GSE17536 set, *p* = 0.013; GSE17537 set, *p* = 0.033). The validation results were consistent with the previous construction result (Fig. 6F–H).

### Comprehensive analysis of tumor mutations and clinical relevance in different m6A/m5C/m1A score groups

**Fig. 7:**
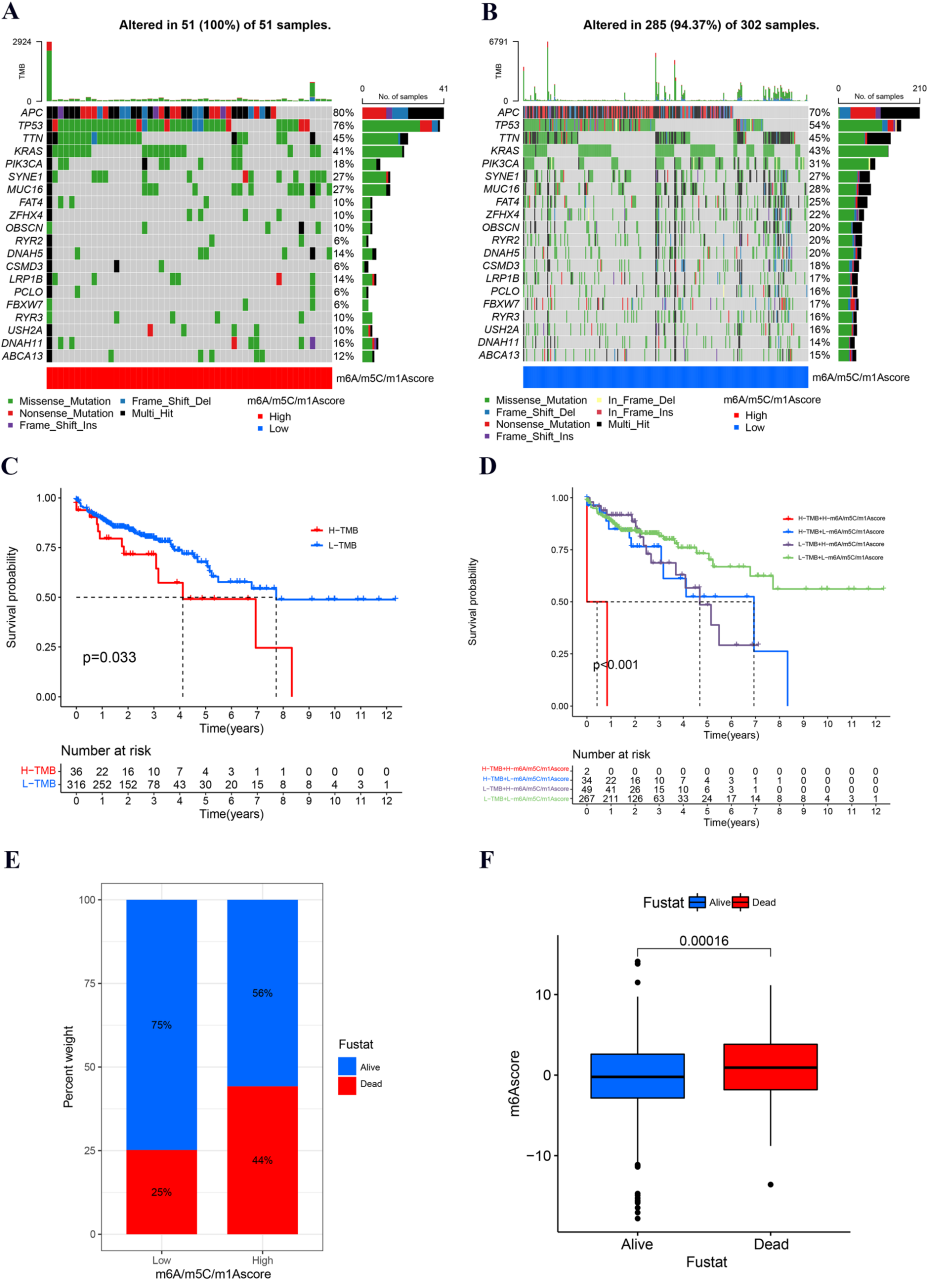
Comprehensive analysis of tumor mutations and clinical relevance in the m6A/m5C/m1A score groups. **A**, **B** Somatic mutation frequency in different score groups. Different colors represent different types of mutations. **C**, **D** Survival analysis of TMB and TMB combined with the m6A/m5C/m1A score. TMB: tumor mutation burden. **E** Rates of survival and death in patients in different score groups. **F** Differential levels of the m6A/m5C/m1A score between surviving and non-surviving patients. Blue and red represent alive and dead patients, respectively

To further assess the clinical value of the m6A/m5C/m1A score, we first performed tumor mutation analysis in different m6A/m5C/m1A score groups. The results indicated that the high score group had higher mutation rates (100% vs. 94.37%). The top five mutated genes in the two score groups were APC, TP53, TTN, KRAS, and PIK3CA, and the top three mutation types were missense mutations, nonsense mutations, and multihit mutations (Fig. 7A, B). Previous studies have illustrated that there is a close connection between TMB and immunotherapy responses. Therefore, we performed survival analysis of TMB, which revealed that patients had a better prognosis in the high TMB group (*p* = 0.033) (Fig. 7C). We further investigated the relationship between the m6A/m5C/m1A score and TMB, and the results indicated that the best survival outcome was observed in the low score and the low TMB group (*p* < 0.001) (Fig. 7D). In addition, we investigated the prognostic value of the score in groups stratified according to different clinical characteristics (age, sex, stage), and the low score group had better survival outcomes in these subgroup analyses (Fig. S3). As shown in Fig. 7E, the death rate of colon cancer patients was higher in the high score group, and rank test results indicated that the m6A/m5C/m1A score was higher in dead patients (Fig. 7F).

### Immunotherapy analysis of colon cancer patients in the m6A/m5C/m1A score groups

**Fig. 8:**
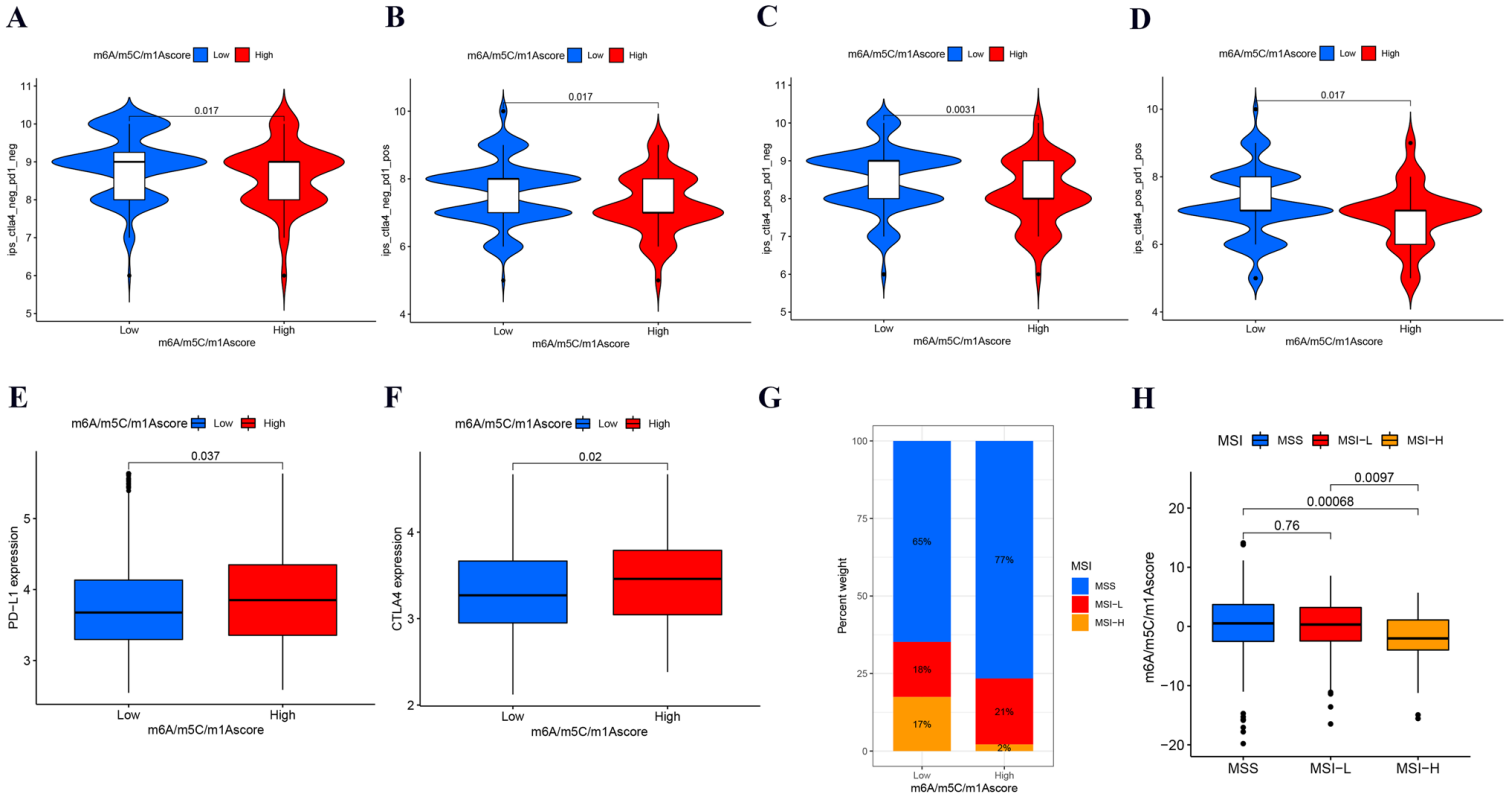
Immunotherapy analysis of colon cancer patients in different m6A/m5C/m1A score groups. **A**–**D** Differential levels of IPS with CTLA-4 (pos/neg)/PD-1 (pos/neg) in different score groups. pos: positive, neg: negative. IPS: immunophenoscore. Blue and red represent low and high expression, respectively. **E**–**F** The expression levels of PD-L1 and CTLA4 in different score groups. **G** Rates of different MSI statuses in different score groups. (H) Differential levels of the m6A/m5C/m1A score in different MSI status groups. *MSS* microsatellite stable, *MSI-H* high microsatellite instability, *MSI-L* low microsatellite instability

Immunotherapy is playing an increasingly main role in the current treatment of colon cancer, especially immuno-checkpoint inhibitor (ICI) treatment, including anti-CTLA-4/anti-PD-L1. Therefore, we first identified the expression levels of PD-L1/CTLA-4 in different score groups, and the results implied that PD-L1/CTLA-4 had higher expression levels in the high score group (Fig. 8E, F). IPS is a novel immunotherapeutic factor, and MSI status is strongly correlated with the response to immunotherapy. We further assessed IPS and MSI status in different score groups to predict immunotherapy response. The IPS in the low score group increased significantly compared to that in the high score group in CTLA-4 (pos/neg)/PD-1 (pos/neg) (Fig. 8A–D). Moreover, we explored the clinical significance of the m6A/m5C/m1A score in groups with different MSI types (MSS, MSI-H, MSI-L). As seen in Fig. 8G, Among patients with high regulator scores, the subgroups with MSS and MSI-L status had higher response rates. Higher m6A/m5C/m1A score were shown for those with MSS compared with those with other MSI types (Fig. 8H).

### Validation of m6A/m5C/m1A regulators at the protein and mRNA expression levels

**Fig. 9:**
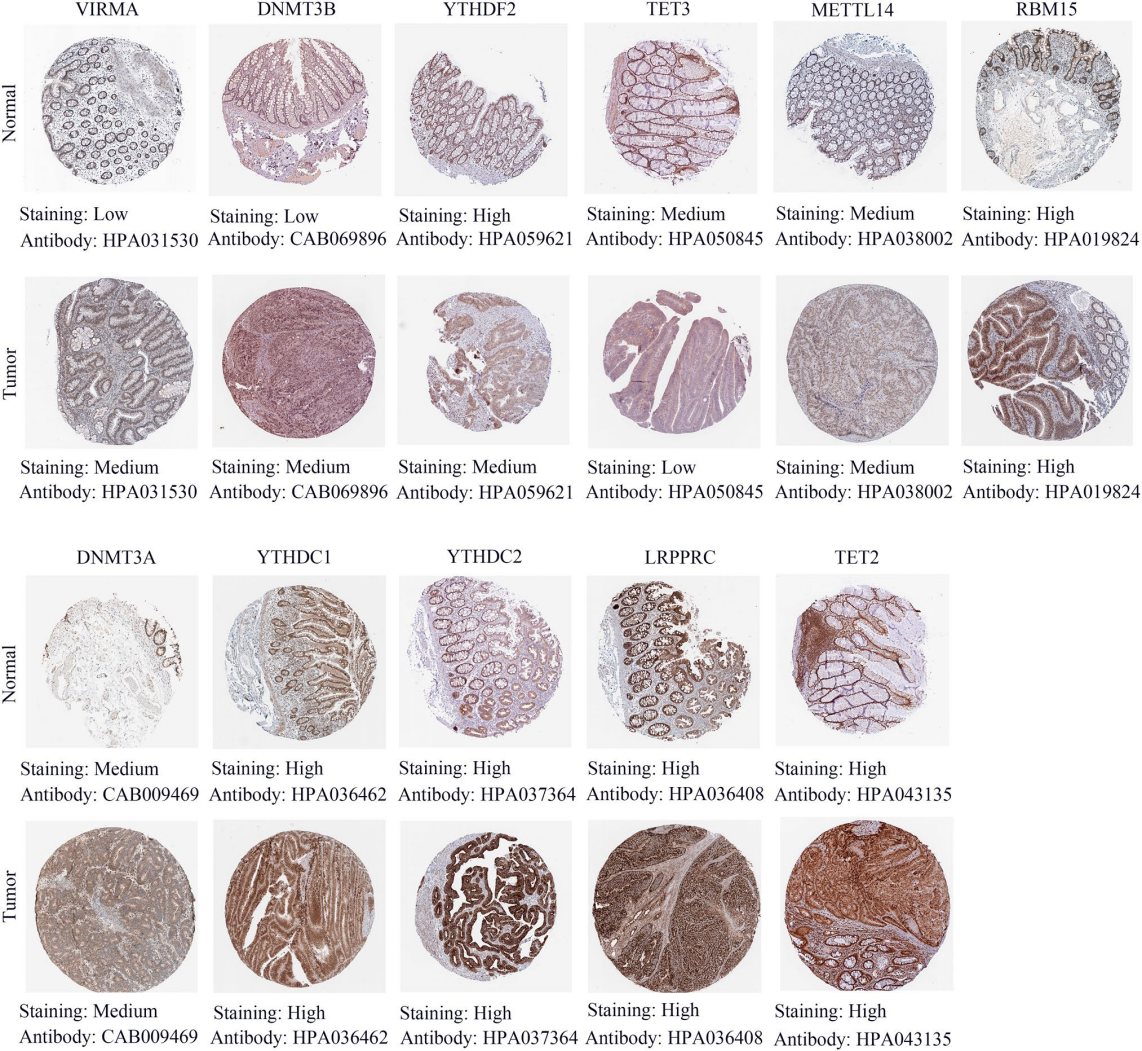
Validation of m6A/m5C/m1A regulators at the protein expression level. Staining degree: high, medium, low, and not detected

**Fig. 10:**
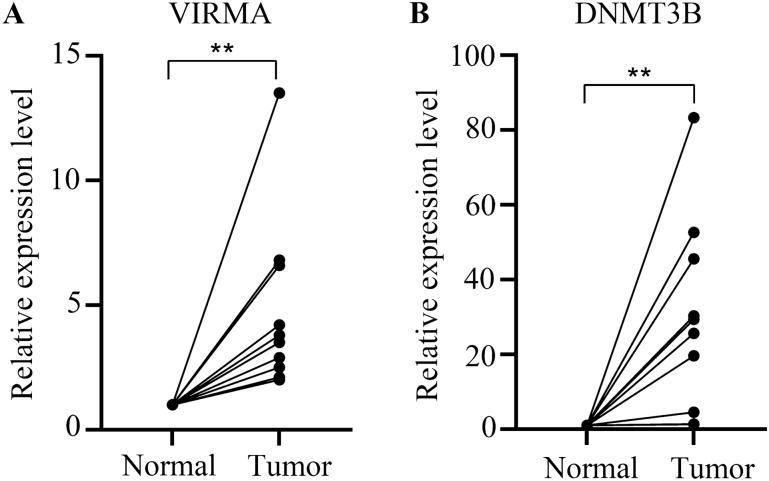
Validation of hub m6A/m5C/m1A regulators at the mRNA expression level. *p < 0.05, **p < 0.01, ***p < 0.001

To investigate the key biological function of m6A/m5C/m1A regulators in colon cancer, we first assessed the protein expression levels of m6A/m5C/m1A regulators in colon cancer tissues and normal tissue with immunohistochemical staining data from the HPA. The results indicated that VIRMA and DNMT3B were significantly higher in colon cancer tissue, which was consistent with the previous analysis (Fig. 9). However, the protein levels of YTHDF2 and TET3 were different from the results of the previous analysis. The rest of the regulators showed no difference in tumor and normal tissues. YTHDF3 protein expression did not correspond to the staining outcome. Next, we further explored the mRNA expression levels of VIRMA and DNMT3B in 10 pairs of colon cancer and normal tissues with RT-qPCR. The results indicated that VIRMA and DNMT3B had higher mRNA expression levels in colon cancer tissue (Fig. 10), which was consistent with the protein level results. Therefore, we identified that VIRMA and DNMT3B could play vital roles in colon cancer.

## Discussion

Previous reports have found that patients with colon cancer have a high mortality rate, mainly due to limitations of current clinical scoring systems and a lack of individualized treatment (Liu et al. 2022b). At the molecular level, inter- and intratumor heterogeneity contribute to a poor prognosis in patients. Epigenetics play a crucial role in the heterogeneity of colon cancer. Recently, as an important component of epigenetics, RNA modifications, including m6A, m5C, and m1A, have been demonstrated to be involved in various biological processes (Jung et al. 2020). RNA modifications interact with each other and play a joint role in the progression of cancers. However, many studies have focused solely on single RNA modifications, and different RNA modifications have also not been well studied in the tumor microenvironment and immune infiltration. Consequently, exploring overlapping RNA modifications, including m6A, m5C, and m1A, may help elucidate the features of the tumor microenvironment and immune infiltration, aiding in the development of an accurate and convenient clinical scoring system to help diagnose and individualize the treatment of patients with colon cancer.

In our study, we first analyzed the mutation traits, CNV status, gene location, expression levels, and interaction relationships of 48 m6A/m5C/m1A regulators in colon cancer. The waterfall chart showed that ZC3H13 had the highest mutation rate compared with the other regulators. Previous studies have also demonstrated that ZC3H13 may be the main regulator of the Ras-ERK signaling pathway (Zhu et al. 2019). Moreover, CNV mutation analysis implied that the majority of m6A/m5C/m1A regulators showed widespread CNV amplification and that TRMT6 had the highest rate of copy number loss. Gene copy number abnormalities may reveal therapeutic targets or markers of drug resistance in colon cancer (Pos et al. 2021). TRMT6/TRMT61A has been identified to increase m1A methylation of tRNA to increase PPARδ translation, which ultimately drives hepatic cancer stem cell self-renewal and tumorigenesis (Wang et al. 2021). In addition, we further investigated the expression levels of m6A/m5C/m1A regulators in colon cancer and found that there were 12 m6A/m5C/m1A regulators that showed a statistically significant difference between normal and tumor tissues. The above results revealed that m6A/m5C/m1A are involved in the occurrence and progression of colon cancer.

Considering the different biological properties of these regulators, three different clusters were identified with unsupervised cluster analysis. M6A/m5C/m1A cluster A had a poorer survival outcome than the other clusters. However, m6A/m5C/m1A cluster A had higher levels of immune cell infiltration than the other clusters. The main reason may be metabolic reprogramming. Colon cancer cells secrete various factors, including cytokines and chemokines, to reshape their microenvironment, which contributes to the reprogramming of the surrounding immune cells. Eventually, the microenvironment promotes tumor growth and metastasis. For example, myeloid-derived suppressor cells (MDSCs) are mainly enriched in m6A/m5C/m1A cluster C. MDSCs, as immunosuppressive cells, have angiogenic potential and can promote the growth and development of tumors (Motz and Coukos 2011).

To further explore the clinical and biological traits of the m6A/m5C/m1A clusters, three types of gene clusters were identified. The advantage of secondary clustering was that the number of differential genes could be expanded, the differential genes associated with prognosis could be screened, and laid the foundation for building a scoring system. Then, GO results indicated that the DEGs mainly enriched in ribonucleoprotein complex biogenesis, nuclear envelope, and single stranded RNA binding, while KEGG results indicated that the DEGs mainly enriched in nucleocytoplasmic transport, which further validated the role of RNA methylation in the tumor microenvironment.

Although m6A/m5C/m1A regulators played a vital role in regulating prognosis and immune infiltration, due to the differential expression and complexity of m6A/m5C/m1A regulators, the above analysis referred to the regulators as a whole rather than individual regulators. We constructed a m6A/m5C/m1A scoring system to evaluate the clinical value and treatment effect of m6A/m5C/m1A regulators in individuals. First, the m6A/m5C/m1A score could act as a significant predictive factor for colon cancer. As we expected, m6A/m5C/m1A cluster B/C and gene cluster A/C mainly corresponded to low m6A/m5C/m1A scores, which indicated a better prognosis. Next, the prognostic value of the score was validated in three independent cohorts (GSE29621 set, GSE17536 set, GSE17537 set). The results showed that the low m6A/m5C/m1A score group had a better survival outcome, while the high score group had a poorer survival outcome. The validation results were consistent with the previous construction result. Moreover, immune infiltration analysis indicated that the infiltration of activated CD4 T cells, eosinophils, plasmacytoid dendritic cells, and type 2T helper cells was positively related to the m6A/m5C/m1A score, and the score may identify the tumor immunophenotype and guide the use of targeted immunotherapy. In addition, TMB has recently been shown to correlate with clinical outcomes in a variety of cancers, such as melanoma, lung cancer, and colorectal cancer. Several studies have shown that high TMB is effective in predicting objective remission rates and progression-free survival, but TMB has limited ability to predict overall survival (Li et al. 2020). Our work identified that there was a close correlation between TMB and the m6A/m5C/m1A score, and a consolidated forecasting signature including TMB and the score could provide better survival prediction, which further guides immunotherapy. Immunotherapy has quickly replaced other traditional treatment modalities for a number of solid tumors, including colorectal cancer (Ganesh et al. 2019). Following the first success of melanoma treatment, immune checkpoint inhibitors, including anti-PD-L1 and anti-CTLA-4, now play a more critical role in immunotherapy. We found that there was a significant difference in the expression of PD-L1 and CTLA-4 in the different score groups. The IPS indicated better treatment results in the low score group regardless of the status of PD-1 and CTLA-4, which provides a guideline for immunotherapy.

Finally, to identify the key biological function of m6A/m5C/m1A regulators in colon cancer, we performed immunohistochemical staining and RT-qPCR to screen two key biomarkers (VIRMA and DNMT3B). Previous reports have revealed that VIRMA-directed m6A modification promotes NSCLC progression through m6A-dependent degradation of DAPK3 and that VIRMA could become a new therapeutic target for NSCLC (Xu et al. 2021). Moreover, VIRMA downregulation attenuated the aggressive phenotype of prostate cancer cells by reducing the stability and abundance of oncogenic lncRNAs through an overall reduction in m6A levels (Barros-Silva et al. 2020). However, the role of VIRMA in colon cancer still needs to be further explored. Moreover, low miR-203 expression in colorectal cancer leads to ABCG2 promoter methylation and significantly reduced expression by attenuating the inhibition of DNMT3B (To et al. 2017), which was consistent with our results.

We constructed and validated an accurate and simple scoring signature to assess the survival outcomes and immune infiltration characteristics of colon cancer patients. There are some limitations to our study. First, all sample data in this study are retrospective and require further validation with data from a multicenter and prospective study. Second, some clinical and molecular feature datasets are very inadequate, which may mask potential associations between the m6A/m5C/m1A score and certain variables. Third, the role of many regulators in colon cancer is unknown and needs to be further validated with in vivo and in vitro experiments.

## Conclusion

We constructed and validated a stable and powerful m6A/m5C/m1A score signature to assess the survival outcomes and immune infiltration characteristics of colon cancer patients, which further guides the optimization of personalized treatment, making it valuable for clinical translation and implementation.

## Supplementary Information

Below is the link to the electronic supplementary material.

Supplementary file1 Fig. S1: The overall survival curves of colon cancer patients treated with m6A/m5C/m1A regulators. (A-G) High levels of 7 m6A/m5C/m1A regulators were associated with a favorable prognosis. Red and blue represent high and low expression, respectively (TIF 1768 KB)

Supplementary file2 Fig. S2: The levels of tumor-infiltrating immune cells and immune functions in different m6A/m5C/m1A clusters (TIF 2353 KB)

Supplementary file3 Fig. S3: (A-F) Prognostic value of the m6A/m5C/m1A score in groups stratified by different clinical characteristics (age, sex, stage) (TIF 1254 KB)

Supplementary file4 (XLSX 1298 KB)

Supplementary file5 (XLSX 11 KB)

## Data Availability

All transcriptome and expression profile data were obtained from The Cancer Genome Atlas and Gene Expression Omnibus. These data are publicly available.
