# Supplementary figures and images for: A novel m6A/m5C/m1A score signature to evaluate prognosis and its immunotherapy value in colon cancer patients
Source: J Cancer Res Clin Oncol. 2023 Jul 8;149(13):11995–2012. doi: 10.1007/s00432-023-05033-1 (PMC10465387; doi:10.1007/s00432-023-05033-1)

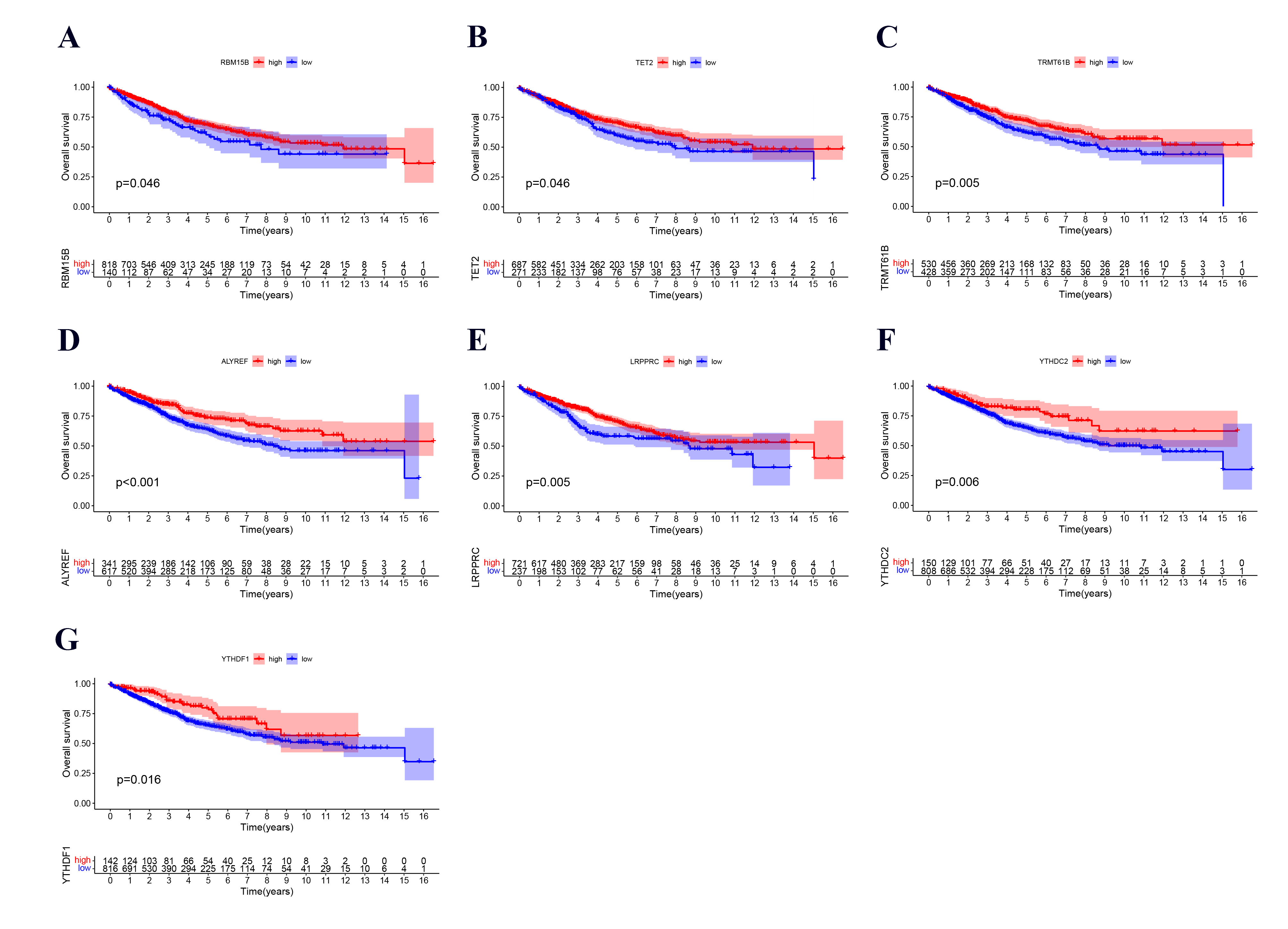

Supplement: Supplementary file 1 — Supplementary file1 Fig. S1: The overall survival curves of colon cancer patients treated with m6A/m5C/m1A regulators. (A-G) High levels of 7 m6A/m5C/m1A regulators were associated with a favorable prognosis. Red and blue represent high and low expression, respectively (TIF 1768 KB) [file 432_2023_5033_MOESM1_ESM.tif]

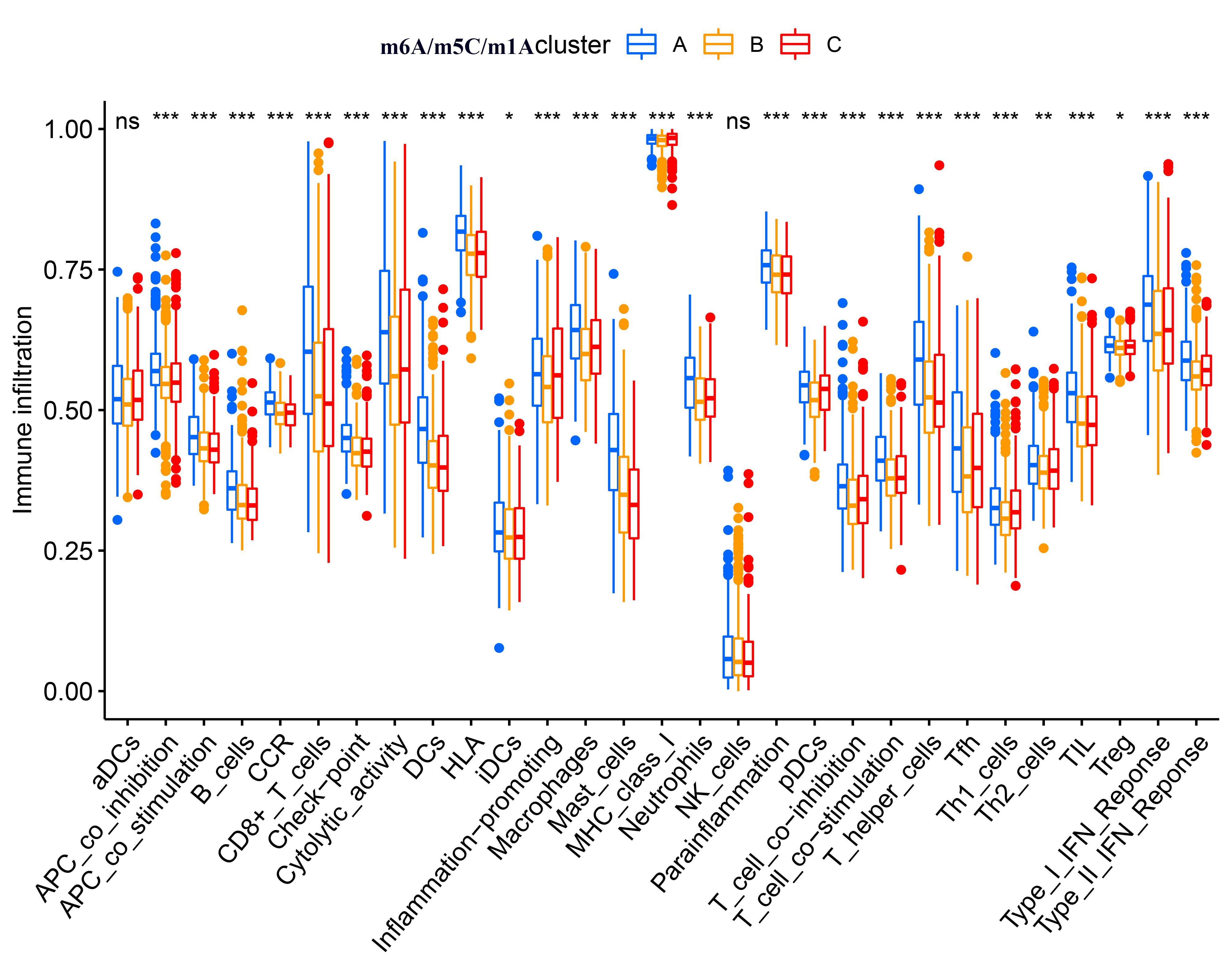

Supplement: Supplementary file 2 — Supplementary file2 Fig. S2: The levels of tumor-infiltrating immune cells and immune functions in different m6A/m5C/m1A clusters (TIF 2353 KB) [file 432_2023_5033_MOESM2_ESM.tif]

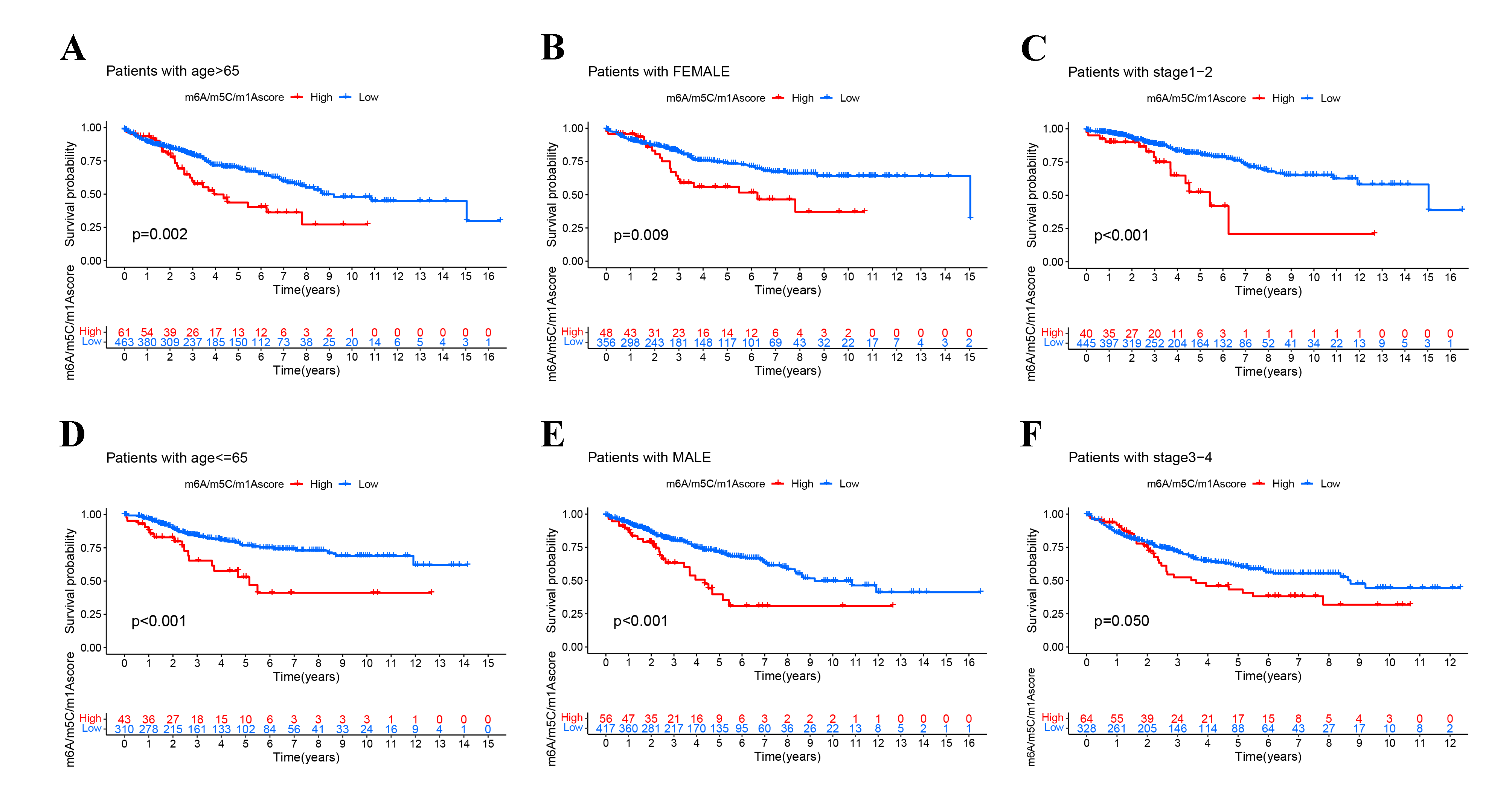

Supplement: Supplementary file 3 — Supplementary file3 Fig. S3: (A-F) Prognostic value of the m6A/m5C/m1A score in groups stratified by different clinical characteristics (age, sex, stage) (TIF 1254 KB) [file 432_2023_5033_MOESM3_ESM.tif]
